# Supplementary material for: A Comparative Longitudinal Study Analyzing Vaginal Microbiota Differences Between Term and Preterm Pregnancies in Korean Women
Source: Medicina (Kaunas). 2025 Apr 18;61(4):752. doi: 10.3390/medicina61040752 (PMC12028669; doi:10.3390/medicina61040752)
Supplement: Supplementary file 1 [file medicina-61-00752-s001.zip › medicina-3541783-supplementary.pdf]

Table S1. Information of primers and probes

No.	Bacterial Species	Target Gene	Oligo name	Length (bp)	Filter Tm (℃)	GC (%)	Fluorescence	Amplicon Size (bp)
1	*L. crispatus*	gyrB	Forward	24	57.6	37.5	-	154
			Reverse	23	58.7	34.8	-	
			Probe	31	69.3	38.7	FAM	
2	*L. iners*	cpn60	Forward	23	57.8	39.1	-	138
			Reverse	22	58.3	50	-	
			Probe	32	66.8	34.4	CY5	
3	*L. gasseri*	cpn60	Forward	22	57	40.9	-	209
			Reverse	22	57.6	50	-	
			Probe	32	68.2	34.4	FAM	
4	*L. jensenii*	cpn60	Forward	21	58.1	47.6	-	138
			Reverse	25	58.3	32	-	
			Probe	31	68.1	41.9	JOE	
5	*W. koreensis*	recN	Forward	24	58.1	29.2	-	117
			Reverse	23	57.2	39.1	-	
			Probe	27	69.4	44.4	JOE	
6	*U. urealyticum*	Urease	Forward	26	58.4	42.3	-	119
			Reverse	24	57.7	37.5	-	
			Probe	28	68.9	35.7	FAM	
7	*U. parvum*	Urease	Forward	23	59	39.1	-	122
			Reverse	22	58.1	40.9	-	
			Probe	31	68.6	35.5	JOE	
8	*G. vaginalis*	Sialidase A	Forward	24	59.6	37.5	-	153
			Reverse	24	57.9	37.5	-	
			Probe	30	70.3	36.7	CY5	
9	*B. fragilis*	gyrB	Forward	23	58.1	43.5	-	154
			Reverse	27	57.4	37	-	
			Probe	26	69.1	46.2	CY5	
10	*P. bivia*	mdsC	Forward	25	57.9	32	-	123
			Reverse	24	58	41.7	-	
			Probe	28	68.9	46.4	FAM	
11	*P. salivae*	rpoB	Forward	24	57.5	41.7	-	168
			Reverse	23	58.2	47.8	-	
			Probe	31	68.3	35.5	CY5	

12	*P. amnii*	rpoB	Forward	25	58.3	36	-	101
			Reverse	22	59.2	54.5	-	
			Probe	27	69.6	44.4	JOE	
13	*Internal Control*	Cesa3	Forward	22	58.4	45.5	-	221
			Reverse	21	59.2	47.6	-	
			Probe	31	70	35.5	TexasRed	

Table S2. Strains used for specificity tests

No.	Microbe	Bank No.
1	*Lactobacillus crispatus*	ATCC 33820
2	*Weisella koreensis*	KACC 17870
3	*Lactobacillus iners*	ATCC 55195
4	*Ureaplasma urealyticum*	ATCC 33699
5	*Ureaplasma pavum*	ATCC 27815
6	*Gardnerella vaginalis*	ATCC 14019
7	*Bacteroides fragilis*	ATCC 25285
8	*Prevotella bivia*	DSM 20514
9	*Prevotella amnii*	JCM 14753
10	*Prevotella salivae*	DSM 15606
11	*Lactobacillus gasseri*	ATCC 3323
12	*Lactobacillus jensenii*	KCTC 5194
13	*Lactobacillus vaginalis*	ATCC 49540
14	*Lactobacillus reuteri*	ATCC 23272
15	*Lactobacillus acidophilus*	KCTC 3145
16	*Lactobacillus coleohominis*	KCTC 21007
17	*Prevotella disiens*	ATCC 29426
18	*Staphylococcus epidermidis*	KCTC 13171
19	*Staphylococcus saprophyticus* subsp. *saprophyticus*	KCTC 3345
20	*Staphylococcus warneri*	ATCC 27836
21	*Streptococcus intermedius*	ATCC 27335
22	*Streptococcus gallinaceus*	KCTC 3876
23	*Streptococcus oralis*	KCTC 5671
24	*Streptococcus downei*	ATCC 33748
25	*Anaerococcus vaginalis*	KCTC 15028
26	*Anaerococcus lactolyticus*	ATCC 51172
27	*Anaerococcus tetradius*	ATCC 35098
28	*Atopobiumvaginae*	ATCC BAA-55
29	*Actinomyces urogenitalis*	KCTC 5117
30	*Peptostreptococcus anaerobius*	ATCC 27337
31	*Proteus mirabilis*	ATCC 29906
32	*Peptoniphilus duerdenii*	KCTC 15408
33	*Dermabacter vaginalis*	KCTC 39585
34	*Enterobacter cloacae* subsp. *cloacae*	ATCC 13047
35	*Enterococcus faecalis*	ATCC 19433
36	*Candida albicans*	ATCC 11006
37	*Candida dubliniensis*	KCTC 17427

38	*Candida glabrata*	ATCC 2001
39	*Candida parapsilosis*	ATCC 22019
40	*Candida tropicalis*	ATCC 750
41	*Weissella cibaria*	KCTC 3807

Figure S1. Graphs of standard curves

<table>
<tr><td>Lactobacillus crispatus</td><td>Weissella Koreensis</td><td>Lactobacillus iners</td></tr>
<tr><td>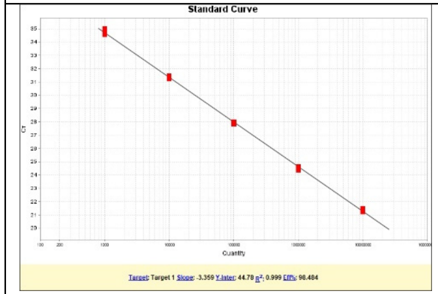
</td><td>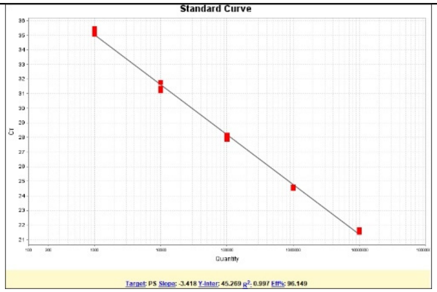
</td><td>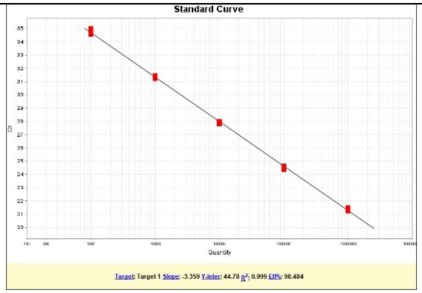
</td></tr>
<tr><td>$R^2 = 0.999$
PCR efficiency=98.484%</td><td>$R^2 = 0.999$
PCR efficiency=99.627%</td><td>$R^2 = 0.998$
PCR efficiency=96.865%</td></tr>
<tr><td colspan="3"></td></tr>
<tr><td>Ureaplasma urealyticum</td><td>Ureaplasma parvum</td><td>Gardnerella vaginalis</td></tr>
<tr><td>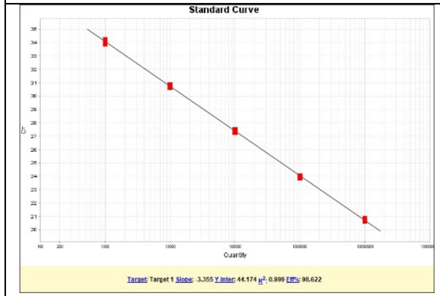
</td><td>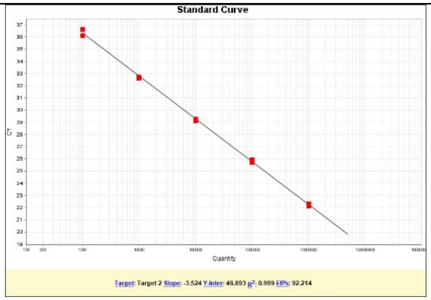
</td><td>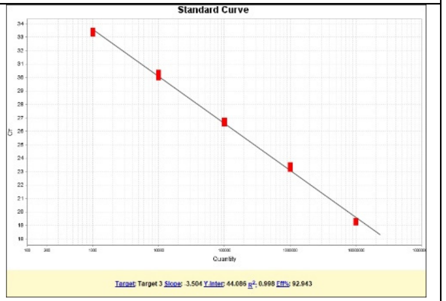
</td></tr>
<tr><td>$R^2 = 0.999$
PCR efficiency=98.622%</td><td>$R^2 = 0.999$
PCR efficiency=92.214%</td><td>$R^2 = 0.998$
PCR efficiency=92.943%</td></tr>
<tr><td colspan="3"></td></tr>
</table>

<table>
<tr><td>Lactobacillus gasseri</td><td>Lactobacillus jensenii</td><td>Bacteroides fragilis</td></tr>
<tr><td>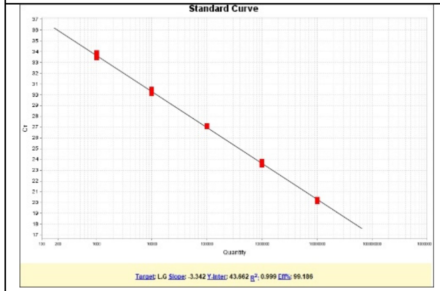
</td><td>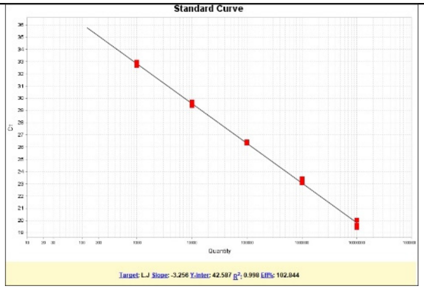
</td><td>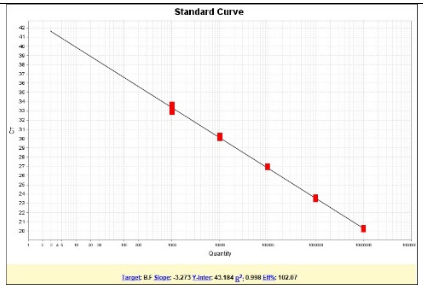
</td></tr>
<tr><td>$R^2 = 0.999$
PCR efficiency=99.186%</td><td>$R^2 = 0.998$
PCR efficiency=102.844%</td><td>$R^2 = 0.998$
PCR efficiency=102.07%</td></tr>
<tr><td></td><td></td><td></td></tr>
<tr><td>Prevotella bivia</td><td>Prevotella amnii</td><td>Prevotella salivae</td></tr>
<tr><td>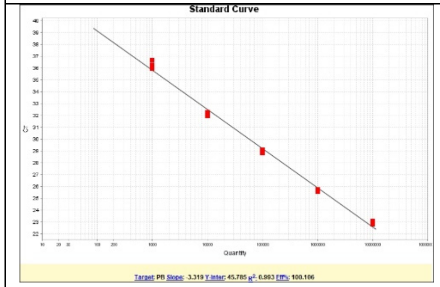
</td><td>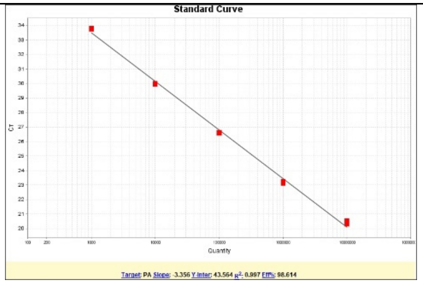
</td><td>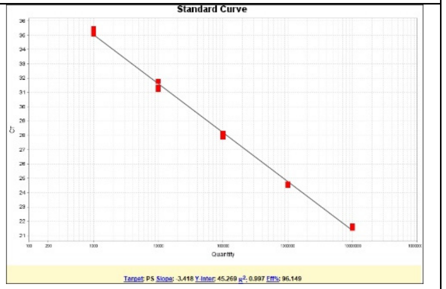
</td></tr>
<tr><td>$R^2 = 0.993$
PCR efficiency= 100.106%</td><td>$R^2 = 0.997$
PCR efficiency= 98.614%</td><td>$R^2 = 0.997$
PCR efficiency= 96.149%</td></tr>
</table>
